# Root Growth, Water and Nitrogen Use Efficiencies in Winter Wheat Under Different Irrigation and Nitrogen Regimes in North China Plain

**DOI:** 10.3389/fpls.2018.01798

**Published:** 2018-12-05

**Authors:** Weixing Liu, Jiarui Wang, Chenyang Wang, Geng Ma, Qiongru Wei, Hongfang Lu, Yingxin Xie, Dongyun Ma, Guozhang Kang

**Affiliations:** ^1^College of Agronomy, Henan Agricultural University, Zhengzhou, China; ^2^State Key Laboratory of Wheat and Maize Crop Science, Henan Agricultural University, Zhengzhou, China; ^3^National Engineering Research Centre for Wheat, Henan Agricultural University, Zhengzhou, China

**Keywords:** winter wheat, irrigation and nitrogen regimes, grain yield, root weight density, water and nitrogen use efficiencies

## Abstract

Excessive nitrogen (N) application combined with water shortage has a negative effect on crop production, particularly wheat (*Triticum aestivum* L.) production in the North China Plain. This study examined root growth and water and nitrogen use efficiencies in wheat grown on loam soil in the North China Plain, from 2012 to 2014 using a fixed-position experiment initiated in 2010. The experiment followed a completely randomized split-plot design with four replications, taking irrigation [no irrigation (W0) versus irrigation at jointing plus flowering (W2)] as the main plot and N treatment (0, 180, 240, and 300 kg N ha^-1^) as the subplot. Compared with W0, W2 increased grain yield and root weight density (RWD) by up to 91.3 and 57.7% in 2012–2013, and 15.5 and 43.0% in 2013–2014, respectively, across all N application rates. Irrigation had no effect on grain water use efficiency (WUE_Y_), but caused a decrease in biomass WUE at vegetative growth stage (WUE_F_) and at grain-filling stage (WUE_M_). Significant improvements in grain yield and biomass WUE during vegetative growth stage, and reductions in nitrogen-use efficiency (NUE) and RWD, were observed with increasing N application. Compared with non-N treatment, N treatment increased yield by up to 98.9 and 93.7% in 2012–2013 and 2013–2014, respectively, decreasing RWD by 12.0 and 16.9%. Correlation analysis further revealed that RWD was positively correlated with grain yield, evapotranspiration (ET) and NUE. NUE was also positively correlated with nitrogen uptake efficiency (UPE). Overall, the findings suggest that optimal N application improves NUE by increasing above–ground nitrogen uptake as a result of optimized RWD and a synchronous increase in WUE, thus increasing yield. Under the experimental conditions, an N application rate of 240 kg N ha^-1^ plus irrigation at jointing and flowering is recommended.

## Introduction

The North China Plain, which covers an area of 3.2 × 10^5^ km^2^, supplies more than 50% of the winter wheat (*Triticum aestivum* L.) produced in China (National Bureau of Statistics of China, 2013). Water is the main limiting factor in primary wheat production (Austin, 2011). In recent years, the water supply has been affected by shortages and over-exploitation of groundwater due to the intensification of agriculture (Li et al., 2008; Zhang et al., 2010). According to Plaut et al. (2004), grain yield, nitrogen absorption, and nitrogen-use efficiency (NUE) decrease under water deficient conditions. Studies have also shown that increasing irrigation rates cause an increase in above–ground nitrogen uptake (AGN), evapotranspiration (ET) and grain yield, but a decrease in water use efficiency (WUE) in wheat (Sun et al., 2006; Zhang et al., 2006). Appropriate irrigation is therefore fundamental in terms of nitrogen absorption, water and nitrogen use efficiencies and increased grain yield (Xu et al., 2005; Sun et al., 2006).

Nitrogen (N) is an essential plant nutrient, affecting productivity, NUE and WUE in wheat (Zhou et al., 2011; Grant et al., 2012; Devadas et al., 2014). Excessive N application not only causes a waste of resources and economic losses, but can also have an adverse effect on the environment. N application has been shown to increase leaching of NO_3_–N in the North China Plain (Ju and Christie, 2011) and in irrigated Mediterranean areas (Lloveras et al., 2001). Although excessive N application increases grain yield, it causes a decrease in NUE and is not conducive to the use of deep soil water during wheat growth cycle (Chen et al., 2006; Zhao et al., 2006; Foulkes et al., 2009; Ma et al., 2009). Understanding the optimal rate of N fertilizer application is therefore important not only in terms of efficient root growth and distribution, but also water storage and utilization, and thereby, NUE and WUE in winter wheat (Luis et al., 2005; Sepaskhah et al., 2006; Rasmussen et al., 2015).

Although roots contribute to only 10 ∼ 20% of the total plant weight, a well-developed root system is essential for nutrient and water absorption, and therefore, growth and final yield in crops (Amato and Ritchie, 2002; Fageria, 2004; Donssan et al., 2006). Increased capacity for root absorption of water and nutrients is conducive to increased yield and water and nitrogen use efficiencies (Feddes and Raats, 2004; Palta et al., 2007). Root density and depth are strongly influenced by irrigation regimes via the soil water content (Li et al., 2010). In general, slight water shortages during vegetative growth cause an increase in vertical root penetration, decreasing the root length density (RLD) in upper soil layers and increasing the RLD in deeper layers (Zhang and Yang, 2004; Li et al., 2010). However, Xu et al. (2016) suggested that under non-irrigation conditions RLD is lower than that under limited-irrigation in the 0–80 cm soil layer at maturity.

In terms of N application, moderate quantities have been shown to favor root growth in winter wheat (Hedegaard et al., 2006). Furthermore, root density in winter wheat was found to increase with N fertilization up to 150 kg N ha^-1^ (Rasmussen et al., 2015), with higher N rates (200 kg N ha^-1^) causing a reduction in root growth in subsoil layers (Hedegaard et al., 2006). Moreover, Wang et al. (2009) suggested that low availability of N in the soil increases root biomass. Above–ground growth and development, which depends on the acquisition of soil nutrients and water, is therefore closely associated with root morphology and physiology (Ju et al., 2015). Crops with a low root/shoot (R/S) ratio, resulting in partitioning of more dry matter to above–ground biomass, therefore tend to have a higher WUE and grain yield (Zhang X. et al., 2009; Ma et al., 2010). Root/shoot ratio was always higher under water and nitrogen stressed conditions mainly due to lower adverse effect on root growth than on shoot growth (Zhang X. et al., 2009; Ruggiero and Angelino, 2011).

The North China Plain is one of the most important food production regions in China, with wide-spread double cropping of wheat–maize. However, unsuitable levels of irrigation and excess N supplementation are common in this region, causing a decrease in WUE and NUE, thereby increasing production costs and polluting the soil and underground water. We therefore conducted a fixed-position experiment examining the effect of irrigation and nitrogen regimes initiated in 2010 on winter wheat production under high-yield conditions in the North China Plain. The objectives were: (i) to investigate the responses of yield, root growth, and water and nitrogen use efficiencies to irrigation and N application; (ii) to quantify the relationships among root weight density (RWD), grain yield and water and nitrogen use efficiencies; and (iii) to determine the optimal irrigation and N regime for the region.

## Materials and Methods

### Experimental Site and Weather Conditions

A fixed-position experiment of irrigation and N regimes in the same experimental plot was implemented in October 2010 in a wheat–maize rotation system on the research farm of Henan Agriculture University, Zhengzhou (113°38′39″ E, 34°47′51″ N), China. The arable layer (0–20 cm) had a loam texture (32.3% sand, 57.7% silt, 10.0% clay) with a tendency toward sandy-loam in deeper layers (>1.5 m), a field capacity of 23.9% volumetric soil water and a bulk density of 1.28 g cm^-3^, and the amounts of organic matter, total nitrogen, available phosphorus and available potassium were 17.47, 0.84 g kg^-1^ and 18.83, and 212.56 mg kg^-1^.

**FIGURE 1:**
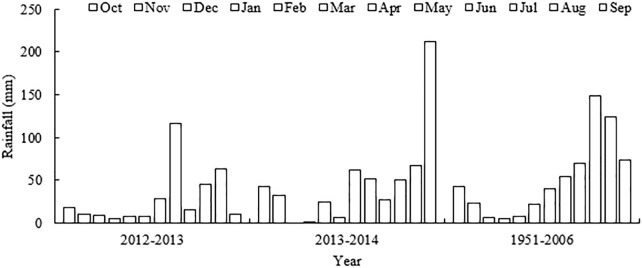
Annual precipitation by month (October to September) during the wheat-maize growing seasons from 2012 to 2014 and the corresponding monthly averages from 1951 to 2006 at the experimental site.

The climate is typical continental monsoon: rainy and hot in summer, dry and cold in winter, with an annual mean temperature of 14.3°C and annual precipitation of 617.1 mm (1951–2006), an average of 201.2 mm falling during the wheat growing season. While it was 22.3, 40.1, and 53.7 mm in March, April, and May, respectively. Data was collected in wheat growing seasons in 2012–2013 and 2013–2014, 2 years after initiation of the experiment. The rainfall showed an uneven distribution pattern, which was mainly concentrated in the late stage of wheat growth. The total rainfall in April and May accounted for 70.8 and 51.7% of the total rainfall during the wheat growing seasons in 2012–2013 and 2013–2014, respectively (Figure 1). Compared to the 2013–2014 growing season, 2012–2013 was relatively dry, with only 13.1 mm rainfall from jointing to flowering (March 10 to April 15), which was only about 30% compared to long term average. In contrast, 100.6 mm occurred in late May, 54.0 mm higher than the long-term average and an adverse event in terms of wheat production (causing early senescence).

### Experimental Design and Crop Management

The experimental design combined two irrigation regimes and four N rates. Plots were arranged in a split-plot design with four replicates. Main plots were assigned to irrigation: no irrigation water applied after sowing (W0), and irrigation at jointing and flowering (W2). During the 2013–2014 wheat growing season, uniform post-sowing irrigation (50 mm) was applied to all plots, regardless of treatment, to ensure proper germination and seedling establishment. During the maize growing season in 2012, irrigation was applied at the V12 stage under W2 treatment, while in 2013, all treatments (including W0) were irrigated after sowing, and W2 treatment was irrigated at the tasseling stage. Irrigation was applied using a movable sprinkler system, with application of 75 mm each time as measured with a water meter. Subplots were treated with four nitrogen rates: 0, 180, 240, and 300 kg N ha^-1^ during the wheat growing season and 0, 225, 300, and 375 kg N ha^-1^ during the maize growing season in the same experimental plot, and designated N0, N180, N240, and N300, respectively.

Summer maize was grown prior to planting of winter wheat, with all straw returned to the soil before tillage in both growing seasons. Phosphorus was applied as triple superphosphate (P_2_O_5_, 46%) at a rate of 326 kg ha^-1^ and potash as potassium chloride (K_2_O 60%) at a rate of 200 kg ha^-1^. Half the N (urea, 46% N), and all the phosphorus and phosphate were applied before plowing at the time of sowing. The remaining N was applied at the jointing stage in each N treatment. Each experimental plot was 7 m in length and 2.9 m in width. The local wheat cultivar Yumai 49–198 was used, with seeds sown in a row spacing of 20 cm at 240 kg ha^-1^ on October 12 in both growing seasons. The seeding rate was adjusted for a density of 220–240 seeds m^-2^. Wheat was harvested on May 23 and 25 under N0 treatment, and on May 26 and 29 under N treatment in 2013 and 2014, respectively. Maize was planted immediately after wheat harvest. P_2_O_5_ was applied at 90 kg ha^-1^ and K_2_O at 120 kg ha^-1^, with 30% of the nitrogen in each treatment applied at the maize elongation stage. The remaining 70% was applied at the V12 stage.

Additional protective measures were taken to ensure healthy growth such as the spraying of insecticides, fungicides, and herbicides. Accordingly, no significant incidence of pests, diseases, or weeds was observed in any of the treatment sites in either growing season.

### Sampling and Measurements

A quadrat of 8.7 m^2^ was harvested during both growing seasons. After air-drying, the samples were threshed and the grain yield (GY) was weighed. Sub-samples of approximately 500 g of air-dried grain were oven-dried to estimate the grain water content. The GY for each plot was adjusted to a standard 12% moisture content. An additional 30 single stems per treatment were sampled for calculation of N accumulation in different organs. Samples were separated into leaves, stems + sheaths, grains, and ear rachises + glumes. Separated samples were oven–dried at 70°C to a constant weight then milled and analyzed for N concentration using the semi-micro Kjeldahl method (Bao, 2000). N accumulation was determined by multiplying the N concentration by the dry weight.

#### Root Sampling

Roots were sampled at jointing, flowering, and maturity by removing soil blocks. Briefly, blocks were dug at 20-cm intervals to a vertical depth of 100 cm with five separated layers, 0–20, 20–40, 40–60, 60–80, and 80–100 cm. Before root sampling, corresponding above–ground parts were sampled for calculation of the R/S ratio. Each sampling area was 0.4 m in length (measured perpendicular to the rows and covering plants in two rows) and 0.4 m wide (measured parallel to the rows). Subsequent sampling removed 0.4 m along the original sampling ridge in the same plot. The roots with soil were then transferred to a 100-mesh (0.15 mm) nylon bag and submerged in water for 1 h to wash the soil from the roots. Any remaining soil was then washed from the roots using a low-pressure garden hose, and the clean roots transferred to a sieve (0.25 mm^2^ mesh) suspended in a trough partially filled with water. The roots were collected using forceps and oven–dried at 70°C to determine their dry weight. Root weight density (RWD; g m^-3^), the unit volume of soil root mass, was determined using the following formula:

(1) RWD=M/V,

where M is the total root mass (g) and V is the volume of the soil sample (m^3^).

#### Water Use Efficiency

In each plot, soil cores were therefore obtained at 20-cm intervals to a depth of 160 cm at sowing, flowering, and maturity for the measurement of gravimetric soil water content (θ, g g^-1^). Soil water storage (SWS, mm) was calculated as:

(2) $$SWS=\theta \times \rho_{b}\times h$$

Where, ρ_b_ (g cm^-3^) is the soil bulk density, and h (cm) is the soil thickness. The gravimetric water content (θ, g g^-1^) of the samples was determined by drying them to a constant weight in an oven at 105°C (Bao, 2000).

In addition, ΔS was calculated as by soil water storage in previous stage minus the next stage.

Evapotranspiration (ET; mm) was calculated using the field water balance equation (Yang et al., 2011):

(3) ET=I+P+U−D−R−ΔS,

where I (mm) is the amount of irrigation water applied using the meter, P (mm) is the amount of precipitation measured at the weather station onsite, ΔS (mm) is the change in profile soil moisture. R (mm) is surface runoff (there was no surface runoff, so this variable was ignored). U is the upward capillary flow into the root zone (mm), and D is the downward drainage out from the root zone (mm). Because the groundwater table was approximately 10.2–11.2 m below the soil surface during the 2-year period, capillary flow is negligible (Liu and Wei, 1989). Drainage from the root zone was estimated using the following equation:

$$D=-K(\theta )\cdot \frac{\Delta H}{\Delta Z}\cdot \Delta t$$

Where K (θ) is the unsaturated water conductivity when soil moisture is θ (cm day^-1^), ΔH is the soil water potential between the top-soil and sub-soil (cm), ΔZ is the soil thickness (cm), and Δt is the period of time (in days). Soil water content was measured in the 1.6 m soil profile in this study, since more than 90% of the root system of wheat plants occupies only the first 1.0 m soil layer in the North China Plain, as was shown by several previous studies (Zhang H. et al., 2009; Xu et al., 2016). Therefore, drainage out from the root zone can be ignored in the North China Plain, including at our experimental site (Lv et al., 2011; Xie et al., 2017). Grain water use efficiency (WUE_Y_; kg ha^-1^ mm^-1^) and biomass water use efficiency (WUE_DM_; kg ha^-1^ mm^-1^) were subsequently defined as (Payero et al., 2008; Qiu et al., 2008):

(4) $${WUE}_{Y}=GY/ET,$$

(5) $${WUE}_{F}={DM}_{F/ETF,}$$

(6) $${WUE}_{M}={DM}_{M/ETM,}$$

(7) $${WUE}_{DM}=DM/ET\,$$

where GY (kg ha^-1^) is grain yield, DM_F_, DM_M_ and DM (kg ha^-1^) are above–ground dry-matter during vegetative growth stage, grain-filling, and entire growing season, respectively, and ET_F_, ET_M_, and ET (mm) are evapotranspiration as determined above to represent soil water consumption per plot during vegetative growth stage, grain-filling, and entire growing season, respectively.

#### Nitrogen-Use Efficiency

Wheat absorbs N from the soil and from fertilizer (Barraclough et al., 2010). The other fresh soil samples were then analyzed for soil N_min_ (including NO_3_–N and NH_4_–N). Briefly, 10 g samples of fresh soil sample were placed in 2 M KCL for 1 h. After filtering, the extract solution was then analyzed using a continuous flow analyzer (Bran+Lubbe, Norderstedt, Germany), and the following calculations of N determined (Dai et al., 2013):

NUE: GY per unit N available (from both soil and fertilizer, kg kg^-1^).
Above–ground nitrogen uptake (AGN): total N uptake above–ground per hectare at maturity (kg ha^-1^).
Nitrogen uptake efficiency (UPE): AGN per unit of N available (kg kg^-1^).

#### Photosynthesis Rate

The net photosynthesis rate (Pn) of flag leaves was measured at 7-day intervals from 0 to 28 days after flowering using a Li-6400 portable photosynthesis System (LI-COR, United States), with an artificial light source of 1200 μmol m^-2^ s^-1^. The CO_2_ concentration in the leaf chamber was maintained at 400 μmol mol^-1^ using a CO_2_ injector fitted with a high-pressure liquid CO_2_ cartridge. The temperature was controlled at 25°C, and measurements were taken from 09:00 to 11:00 am.

### Statistical Analysis

Analysis of variance (ANOVA) was performed using SPSS 17.0 software, with comparisons using Duncan’s multiple range tests at α = 0.05 to determine significant differences among treatments. Pearson correlations were used to analyze the relationships among RWD, GY and water and nitrogen use efficiencies. Multivariate analysis of redundancy analysis (RDA) was performed to search for potential relationships among water and irrigation regimes, GY, root weight density, and water and nitrogen use efficiencies (Zhao et al., 2018). Briefly, the experimental indices of each point (GY, RWD, ET, WUE_Y_, WUE_DM_, AGN, and UPE) were dispersion normalization and the RDA analysis was conducted by using vegan package in R language. Finally, the coordinates of each point were obtained.

## Results

### Grain Yield

In the drier year, 2012–2013, the rainfall showed an uneven distribution pattern (Figure 1), and the relatively lower grain yield (GY) was achieved (Table 1). Compared with W0, W2 increased GY by 91.3 and 15.5%, respectively, across all N application rates in 2012–2013 and 2013–2014. As expected, GY also increased with increasing N fertilizer. Under rainfed conditions (W0), the highest GY was observed under N180, with an increase of 94.7 and 95.7% in 2012–2013 and 2013–2014, respectively, compared with N0 treatment. Under irrigation conditions (W2), 240 kg N ha^-1^ (N240) resulted in the highest GY and DM, with an increase of 143.6 and 131.0% compared to N0 in 2012–2013 and 2013–2014, respectively (Table 1). The results also indicate that, among the yield components, the ear number was the most sensitive factor to nitrogen levels and irrigation. Compared to the non-irrigation treatment, irrigation increased the ear number by 49.0% in 2012–2013 and by 13.8% in 2013–2014, respectively. The ear numbers in the N treatment group (vs. the N0 treatment group) were 33.2 and 66.8% higher in 2012–2013 and 2013–2014, respectively (Table 1).

**Table 1:** Effect of irrigation and nitrogen regimes on grain yield and its components in each growing season.

Growing season	Treatment		Ear no. (10^4^ ha^-1^)	Kernels no. (no. ear^-1^)	1000-grain weight (*g*)	Grain yield (kg ha^-1^)
2012–2013	W0	N0	367 d	18.5 d	45.4 a	2055 e
		N1	480 c	26.4 bc	45.0 a	4001 c
		N2	417 cd	25.2 bc	40.1 b	3019 d
		N3	365 d	23.6 c	38.9 b	2902 d
	W2	N0	420 cd	26.4 bc	40.1 b	2952 d
		N1	602 b	28.9 b	40.4 b	6578 b
		N2	705 a	33.9 a	39.2 b	7190 a
		N3	700 a	33.2 a	37.9 b	6187 b
2013–2014	W0	N0	337.5 d	21.2 d	51.0 a	3613.9 d
		N1	610.0 c	29.5 b	47.3 b	7070.8 b
		N2	608.3 c	27.3 c	47.8 b	6773.3 b
		N3	602.5 c	27.3 c	45.5 c	5094.2 c
	W2	N0	331.7 d	28.0 c	47.1 b	3522.5 d
		N1	639.1 b	29.3 b	45.6 c	7168.9 b
		N2	729.9 a	33.4 a	45.1 c	8137.4 a
		N3	699.4 ab	32.9 a	43.7 d	7215.8 b


*Values followed by different letters within each column are significantly different at a probability level of 0.05 within each growing season.*

### Root Weight Density and Its Distribution in Different Soil Layers

**FIGURE 2:**
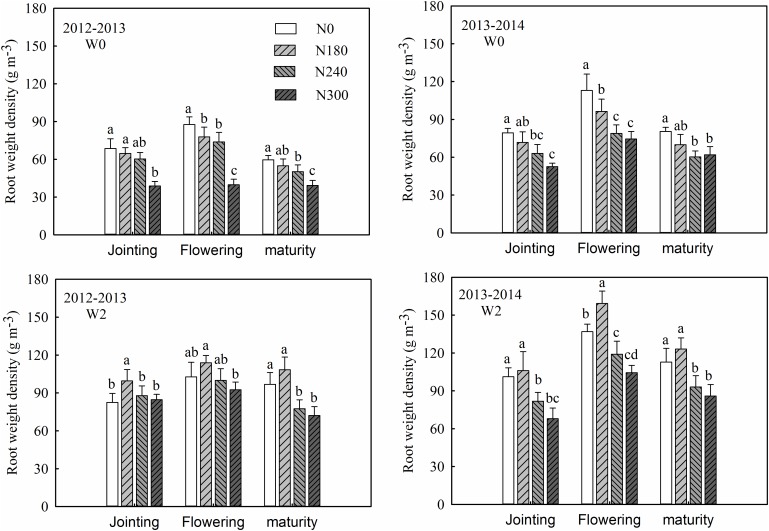
Effects of irrigation and nitrogen regimes on root weight density (RWD) during the 2012–2013 and 2013–2014 growing seasons. Lowercase letters represent statistical significance for one irrigation treatment within each growing season at P_0.05_ using Duncan’s multiple range tests.

Year, irrigation, N application and interactions between irrigation × N and year × irrigation × N had an effect on RWD (Table 2). The RWD in the 100 cm soil profile ranged from 39.75 to 113.85 g m^-3^ in 2012–2013 and 52.61 to 159.27 g m^-3^ in 2013–2014 among the different irrigation and N treatment groups, reaching a maximum at flowering. Compared with flowering, a decrease in RWD of 14.7 and 18.8% was observed at jointing and maturity, in 2012–2013, and of 29.3 and 22.1% in 2013–2014, respectively (Figure 2).

**Table 2:** Variance analysis of grain yield (GY), root weight density (RWD), root to shoot ratio (R/S), evapotranspiration (ET), grain water use efficiency (WUE_Y_), biomass water use efficiency (WUE_DM_) nitrogen use efficiency (NUE), above–ground nitrogen uptake (AGN), and nitrogen uptake efficiency (UPE) in winter wheat according to growing season, irrigation, and nitrogen application.

Factors	GY	RWD	R/S	ET	WUE_Y_	WUE_DM_	NUE	AGN	UPE
Year (Y)	^∗∗^	^∗∗^	^∗∗^	^∗∗^	^∗∗^	^∗∗^	^∗∗^	^∗∗^	^∗∗^
Irrigation (I)	^∗∗^	^∗∗^	^∗∗^	^∗∗^	Ns	^∗∗^	^∗∗^	^∗∗^	^∗∗^
Nitrogen (N)	^∗∗^	^∗∗^	^∗∗^	^∗∗^	^∗∗^	^∗∗^	^∗∗^	^∗∗^	^∗∗^
Y × I	^∗∗^	ns	^∗^	^∗∗^	^∗∗^	ns	^∗∗^	^∗∗^	^∗^
Y × N	^∗∗^	ns	^∗^	ns	^∗∗^	^∗∗^	^∗∗^	^∗^	^∗^
I × N	^∗∗^	^∗∗^	^∗∗^	^∗^	^∗∗^	^∗∗^	^∗∗^	^∗∗^	^∗∗^
Y × I × N	ns	^∗∗^	^∗∗^	ns	ns	^∗∗^	ns	ns	ns


*ns, not significant; ^∗^*P* < 0.05; ^∗∗^*P* < 0.01.*

**FIGURE 3:**
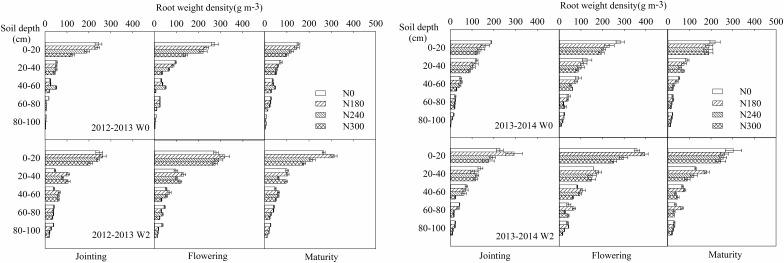
Effects of irrigation and nitrogen regimes on root distribution during the 2012–2013 and 2013–2014 growing seasons.

Irrigation resulted in an increase in RWD in the 100 cm soil profile of 52.5, 46.6, and 73.9% at jointing, flowering and maturity, respectively, in 2012–2013, and 33.7, 43.2, and 52.1%, respectively, in 2013–2014 (Figure 3). RWD decreased with increasing soil depth, the 0–40 cm layer, which plays an important role in wheat growth, accounting for 69.3–89.4 and 71.7–85.6% of the total in 2012–2013 and 2013–2014, respectively. Irrigation also caused an increase in RWD in the 0–40 cm layer of 29.8, 39.0, and 77.2% at jointing, flowering, and maturity in 2012–2013, and 34.9, 45.5, and 47.0% in 2013–2014, respectively. In the drier year, 2012–2013, irrigation increased the proportion of RWD in the deep soil layer (80–100 cm), which was mainly due to the improvement of soil water content in the deep soil layers under irrigation conditions (Wang et al., 2014). However, during normal growing season, irrigation decreased the proportion of RWD in the deep soil layer (80–100 cm), which was slightly lower in W2 (2.5–4.5%) than in W0 (4.1–5.6%) at flowering in 2013–2014 (Figure 3).

Under non-irrigation conditions (W0), N0 treatment produced the highest RWD values, with a gradual decrease with increasing N application. Compared with N0, N treatment resulted in a decrease in RWD in the 100 cm soil profile of 22.3 and 22.7% in 2012–2013 and 2013–2014, respectively (Figure 2), and a corresponding decline in the 0–40 cm layer of 24.4 and 21.5%, respectively (Figure 3). However, under irrigation conditions (W2), highest RWD values were obtained under N180 treatment, with an average decrease in the 100 cm soil profile of 19.9 and 28.9% under N240 and N300 compared with N180 treatment in 2012–2013 and 2013–2014, respectively (Figure 2), and a corresponding decline in the 0–40 cm layer of 20.4 and 23.5%, respectively (Figure 3). Compared with the non-nitrogen treatment (N0), nitrogen treatments, on average, decreased the RWD value by 66.5 and 23.7% in 2012–2013 and 2013–2014, respectively (Figure 3).

### Root-to-Shoot Ratios

**FIGURE 4:**
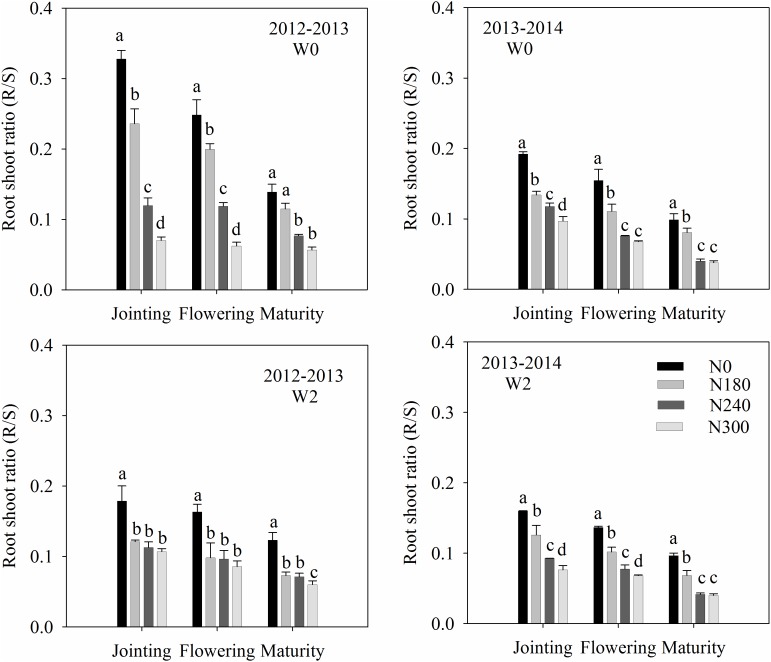
Effects of irrigation and nitrogen regimes on the root to shoot (R/S) ratio during the 2012–2013 and 2013–2014 growing seasons. Lowercase letters represent statistical significance for one irrigation treatment within each growing season at P_0.05_ using Duncan’s multiple range tests.

Root-to-shoot ratios (R/S) decreased gradually with growth, reaching a minimum at maturity (Figure 4). Maximum R/S values were obtained at the jointing stage in both growing seasons. Compared with jointing, a decrease of 15.7 and 43.9% was observed at flowering and maturity in 2012–2013, and 20.4 and 49.5% in 2013–2014, respectively.

R/S was also affected by irrigation and N treatment, decreasing with irrigation and increasing N application. Compared with W0 treatment, a decrease in R/S of 25.4 and 8.8% was observed in 2012–2013 and 2013–2014, respectively. Moreover, compared with N0 treatment, a decrease of 30.1, 47.8, and 59.7% was observed under N180, N240, and N300 in 2012–2013, and 25.8, 46.8, and 53.8% in 2013–2014, respectively (Figure 4).

### Water-Use Efficiency

Analysis of variance showed that both irrigation and nitrogen rate significantly influenced the ET (Table 2). Under W0, ET ranged from 244.5 (2012–2013) to 405.9 mm (2013–2014), and were closely related to the amount of rainfall registered during the two growing seasons. Moreover, irrigation caused a marked increase in ET, ranging from 396.4 to 564.4 mm under W2 (Table 3). Irrespective of the rate, N fertilizer caused an increase in ET in both growing seasons, with averages across years increasing by 27.8, 37.1, and 31.1 mm under N180, N240, and N300 compared with N0, respectively. ANOVA revealed the effect of irrigation on biomass WUE (WUE_DM_); however, there was no effect on grain WUE (WUE_Y_; Table 2). Irrigation caused a decrease in WUE_DM_ of 24.9 and 10.0%, and WUE_F_ of 29.4 and 9.0%, and WUE_M_ 7.5 and 10.8%, across N rates in 2012–2013 and 2013–2014, respectively (Table 3).

**Table 3:** Effect of irrigation and nitrogen regimes on ET between sowing (G_S_) and flowering (G_F_) and between G_F_ and maturity (G_M_), grain WUE (WUE_Y_), biomass WUE at vegetative growth stage (WUE_F_) and at grain-filling stage (WUE_M_) in each growing season.

Growing season	Treatment		ET (mm)			WUE (kg mm^-1^ ha^-1^)			
				
			Total	G_S_-G_F_	G_F_-G_M_	WUE_F_	WUE_M_	WUE_DM_	WUE_Y_
2012–2013	W0	N0	241.5 b	185.8 b	55.7 c	28.4 e	30.6 b	28.9 d	8.5 d
		N180	248.4 b	187.0 b	61.4 b	48.4 b	34.5 a	44.9 b	16.1 b
		N240	244.6 b	182.1 bc	62.5 b	54.1 a	29.7 b	47.8 ab	12.3 c
		N300	243.5 b	180.6 c	62.9 b	55.7 a	30.7 b	49.3 a	11.9 c
	W2	N0	397.1 a	313.0 a	84.1 a	21.8 f	26.4 c	22.7 d	7.4 e
		N180	401.9 a	317.4 a	84.5 a	33.2 d	35.0 a	33.6 c	16.4 b
		N240	398.3 a	315.3 a	83.0 a	38.2 c	30.3 b	36.5 c	18.1 a
		N300	396.4 a	313.5 a	82.9 a	38.5 c	24.3 d	35.5 c	15.6 b
2013–2014	W0	N0	369.3 f	293.0 d	76.3 f	18.8 e	40.9 a	23.4 d	9.8 d
		N180	410.8 e	315.2 c	95.6 d	30.2 c	35.0 b	31.3 bc	17.2 a
		N240	422.2 e	320.8 c	101.4 d	33.7 b	31.7 c	33.2 ab	16.0 a
		N300	421.2 e	318.9 c	102.3 d	37.3 a	30.1 c	35.6 a	12.1 c
	W2	N0	473.3 d	392.2 b	81.1 e	16.6 f	34.6 b	19.7 e	7.4 e
		N180	531.4 c	409.2 ab	122.2 c	28.3 d	34.3 b	29.7 c	13.5 b
		N240	564.4 a	415.1 a	149.3 a	30.5 c	30.4 c	30.5 c	14.4 b
		N300	544.6 b	412.0 a	132.6 b	33.8 b	23.5 d	31.3 bc	13.2 b


*Values followed by different letters within each column are significantly different at a probability level of 0.05 within each growing season.*

Compared to N0, N180 treatment increased WUE_Y_ and WUE_DM_ by an average of 91.2 and 59.3% in 2012–2013, and 69.4 and 48.8% in 2013–2014, respectively (Table 3). The biomass WUE at vegetative growth (WUE_F_) in N treatment was higher than that for N0 by 77.0 and 86.0% in 2012–2013 and 2013–2014, respectively. However, increased N application significantly decreased biomass WUE for grain-filling (WUE_M_). Compared to N180, on average, treatments N240 and N300 decreased WUE_M_ by 13.6 and 20.6% in 2012–2013, and by 10.5 and 22.7% in 2013–2014, respectively.

### NUE and N Accumulation in Different Organs

N_min_ values before wheat sowing in the 100 cm soil profile increased with increasing N application in both growing seasons (Table 4). ANOVA revealed the effect of irrigation, N application and their interaction on NUE and UPE (Table 2). Compared to W0, an increase in NUE of 101.9%, occurred under W2 in 2012–2013; however, an increase of only 20.6% was observed in 2013–2014 (Table 4). With increasing N application from 180 to 300 kg ha^-1^, NUE decreased by 53.8 and 47.4% under both irrigation regimes in 2012–2013 and 2013–2014, respectively, with a corresponding decrease in UPE of 49.0 and 38.9%, respectively. NUE was positively correlated with UPE (*r* = 0.973^∗∗^; Table 6), suggesting that NUE is largely improved through increases in UPE. Both AGN and N accumulation in different organs increased with N application (Tables 4, 5), with an average increase in AGN of 102.3 and 134.1 kg ha^-1^ under N treatment (N180-N300) compared to non-N treatment in 2012–2013 and 2013–2014, respectively. No further increases were observed at N rates exceeding 240 kg N ha^-1^. N distribution was also higher in the stems + sheaths, rachises + glumes and grains under N240, and in the leaves under N300; however, the largest distribution of N in the stems + sheaths, leaves, rachises + glumes and grains were observed with N180 under rainfed conditions in 2012–2013, the drier growing season (Table 5). Correlation analysis also revealed a positive correlation between N accumulation in the leaves and grains and yield, WUE and NUE; however, no correlations were observed with N accumulation in the stems (Table 6).

**Table 4:** Effect of irrigation and nitrogen regimes on N_min_ before sowing, above–ground nitrogen uptake (AGN), nitrogen harvest index (NHI), nitrogen use efficiency (NUE), and nitrogen uptake efficiency (UPE) in each growing season.

Growing season	Treatment		N_min_ (kg ha^-1^)	AGN (kg ha^-1^)	NHI (%)	NUE (kg kg^-1^)	UPE (kg kg^-1^)
2012–2013	W0	N0	59.1 h	54.0 g	63.9 b	–	0.91 a
		N180	118.1 f	143.2 d	63.7 b	13.4 c	0.48 d
		N240	193.8 c	112.4 e	61.1 b	7.0 d	0.26 f
		N300	294.3 a	108.4 e	60.0 b	4.9 e	0.18 g
	W2	N0	94.4 g	72.6 f	69.0 c	–	0.77 b
		N180	131.3 e	191.6 c	68.6 a	21.1 a	0.62 c
		N240	158.8 d	232.1 a	63.5 b	18.0 b	0.58 c
		N300	222.0 b	205.5 b	63.2 b	11.9 b	0.39 e
2013–2014	W0	N0	113.9 g	76.8 f	78.3 a	–	0.67 b
		N180	134.6 e	196.1 c	78.3 a	22.5 ab	0.62 b
		N240	160.3 c	209.2 c	73.2 b	16.9 c	0.52 c
		N300	234.9 a	169.6 d	64.1 e	9.5 e	0.32 e
	W2	N0	89.4 h	84.9 e	74.7 b	–	0.95 a
		N180	127.1 f	208.5 c	70.3 c	23.3 a	0.68 b
		N240	148.4 d	268.2 a	68.8 cd	21.0 b	0.69 b
		N300	191.5 b	237.9 b	66.3 de	14.7 d	0.48 d


*The N_min_ was measured on October 5 in 2012–2013 and on October 6 in 2013–2014, respectively. Values followed by different letters within each column are significantly different at a probability level of 0.05 within each growing season.*

**Table 5:** Nitrogen accumulation (kg ha**^-^**^1^) in different organs at maturity in each growing season.

Growing season	Treatment		Stems + sheaths	Leaves	Rachises + glumes	Grains
2012–2013	W0	N0	9.8*e*	4.5*d*	5.2*e*	34.5*f*
		N180	27.0*b*	12.7*b*	12.3*bc*	91.2*c*
		N240	21.2*c*	11.2*c*	11.3*c*	68.7*d*
		N300	21.3*c*	5.2*d*	11.3*c*	65.0*d*
	W2	N0	11.2*d*	7.0*d*	6.1*d*	50.1.1*e*
		N180	33.3*a*	11.7*c*	15.2*b*	131.4*b*
		N240	36.1*a*	22.4*a*	26.3*a*	147.3*a*
		N300	28.6*b*	23.5*a*	23.5*a*	130.0*b*
2013–2014	W0	N0	9.2*e*	3.3*e*	4.2*e*	60.1*d*
		N180	27.5*c*	7.0*d*	8.0*d*	153.6*b*
		N240	38.0*b*	7.7*d*	10.4*cd*	153.1*b*
		N300	35.1*b*	12.4*c*	13.4*bc*	108.7*c*
	W2	N0	11.9*d*	4.1*e*	5.5*e*	63.4*d*
		N180	32.9*b*	11.6*c*	17.4*a*	146.6*b*
		N240	47.9*a*	17.3*b*	18.6*a*	184.4*a*
		N300	45.5*a*	20.2*a*	14.5*b*	157.7*b*


*Values followed by different letters within each column are significantly different at a probability level of 0.05 within each growing season.*

**Table 6:** Correlation coefficients among grain yield (GY), root weight density (RWD), and water and nitrogen use efficiencies and related parameters.

	GY	JRWD	FRWD	MRWD	ET	WUE_Y_	WUE_DM_	NUE	UPE	SN	LN	RN	GN
JRWD	0.355*												
FRWD	0.420**	0.857**											
MRWD	0.418**	0.841**	0.903**										
ET	0.719**	0.700**	0.683**	0.781**									
WUEY	0.863**	0.011	0.099	0.039	0.292*								
WUEDM	0.558**	-0.262	-0.179	-0.250	-0.037	0.817**							
NUE	0.846**	0.693**	0.758**	0.731**	0.620**	0.780**	0.295						
UPE	-0.034	0.531**	0.577**	0.521**	0.191	-0.185	-0.397**	0.973**					
SN	-0.019	0.030	-0.291*	-0.150	0.118	-0.122	0.117	-0.222	-0.327*				
LN	0.524**	0.062	0.334*	0.289*	0.486**	0.680**	0.380**	0.400**	-0.474**	0.466**			
RN	0.588**	0.198	0.048	0.108	0.522**	0.403**	0.388**	0.158	-0.350**	0.631**	0.244		
GN	0.947**	0.185	0.244	0.235	0.569**	0.895**	0.713**	0.798**	-0.170	0.889**	0.580**	0.624**	
NHI	0.451**	0.240	0.415**	0.270	0.122	0.559**	0.248	0.762**	0.305**	0.070	-0.290*	-0.216	0.428**


*GY, grain yield; JRWD, FRWD and MRWD, root weight density at jointing, flowering and maturity, respectively; ET, evapotranspiration; WUE_*Y*_, grain water use efficiency; WUE_*DM*_, biomass water use efficiency; NUE, nitrogen use efficiency; UPE, nitrogen uptake efficiency; SN, LN, RN and GN, nitrogen accumulation in stems + sheaths, leaves, ear rachises + glumes, and grains, respectively; NHI, nitrogen harvest index. ^∗^p < 0.05; ^∗∗^p < 0.01.*

### Photosynthesis Rate

**FIGURE 5:**
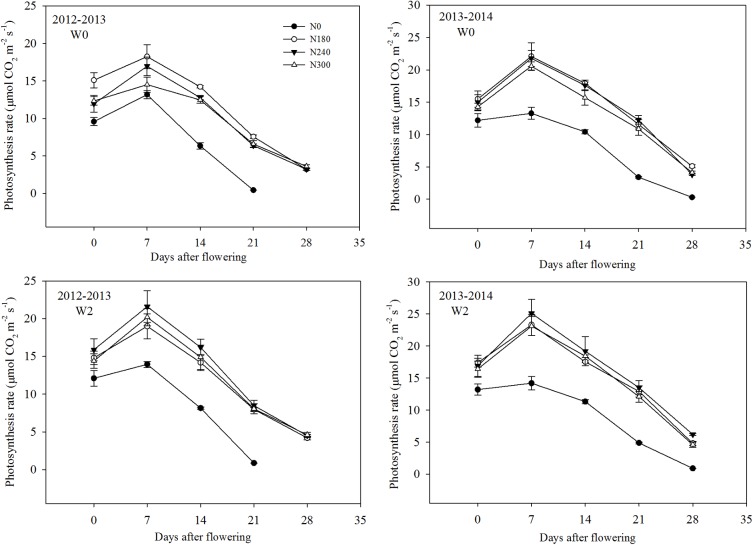
Effect of irrigation and nitrogen regimes on the photosynthesis rate of flag leaves during grain filling during 2012–2013 and 2013–2014 growing seasons.

The photosynthesis rates (Pn) of flag leaves after flowering are shown in Figure 5. Pn under N0 treatment was significantly lower after flowering in both growing seasons. N1 treatment resulted in the highest Pn under W0 conditions during 2012–2013, while N2 treatment resulted in the highest Pn after flowering during the 2013–2014 growing season.

### Redundancy and Correlation Analysis

**FIGURE 6:**
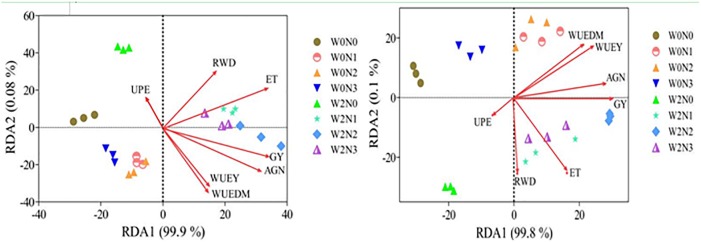
Redundancy analysis (RDA) of the 2012–2013 **(left)** and 2013–2014 **(right)** growing seasons in terms of grain yield, root weight density and water and nitrogen use efficiencies, calculated from RDA of each irrigation and nitrogen regime. GY, grain yield; RWD, root weight density across three stages; ET, evapotranspiration; WUE_Y_, grain water use efficiency; WUE_DM_, biomass water use efficiency; AGN, above–ground nitrogen uptake; UPE, nitrogen uptake efficiency.

RD1 and RD2 explained 99.9 and 0.08% of the overall variance in 2012–2013, and 99.8 and 0.1% of the variance in 2013–2014, respectively (Figure 6). Along RD1, root and water and nitrogen use efficiencies showed significant separation of N0 and N240 and N300 under W0 conditions and N240 and N300 under W2 conditions. Analysis also revealed susceptibility of GY, ET, WUE_Y_, WUE_DM_, AGN, and RWD to irrigation and N regimes, remaining on the right side of the axis in both growing seasons. The RDA plots were interpreted in terms of the Euclidean distances between centroids, and between centroids and individual objects. The angles of the vectors plotted in the plane of the first two RDA axes, which explain the largest proportion of the variation present, show the strength of correlation between response and explanatory variables (a narrow angle indicates a strong correlation; Figure 6). RWD was positively correlated with yield, NUE and ET at all sampling times. The coefficients between RWD and yield, and RWD and NUE were higher at flowering than at jointing and maturity, but lower between RWD and ET. N accumulation in leaves and grains was positively correlated with yield and WUE and NUE (Table 6).

## Discussion

Appropriate irrigation and optimal N application are vital in terms of increased grain yield (Pandey et al., 2001; Paolo and Rinaldi, 2008; Karam et al., 2009; Wang et al., 2010; Li et al., 2015). In this study, grain yield (GY) was higher under irrigation treatment (W2) than non-irrigation treatment (W0) in both growing seasons. Irrigation resulted in an increase in GY of up to 91.3% in 2012–2013, but only 15.5% in 2013–2014 (Table 1), reflecting the importance of irrigation in a dry growing season such as 2012–2013. The wheat yield was also affected by the precipitation during the maize growing seasons, In the growing seasons of 2012 and 2013, the total rainfall was 362.2 and 154.6 mm (Figure 1). In 2013, there was less rainfall in September in 2013 (9.8 mm). Therefore, irrigation was applied to all plots to ensure proper germination and seedling establishment.

Studies in China suggest that the appropriate rate of N application during wheat production ranges from 150 to 225 kg N ha^-1^ depending on the expected yield, soil type, and weather conditions (Wang et al., 2010; Ju and Christie, 2011; Zhong and Shangguan, 2014). In this study, an N application rate of 180 kg N ha^-1^ under rainfed conditions and 240 kg N ha^-1^ under irrigation produced the maximum grain yield, respectively, with additional increases in N having no yield benefits (Table 1). Excessive N rates not only result in a decrease in chlorophyll content and photosynthesis rate, but also prolonged leaf senescence, preventing the transportation of photosynthetic products to grains under dry, hot windy conditions, thereby decreasing yield (Shangguan et al., 2000; Zhong and Shangguan, 2014).

Tilling et al. (2007) demonstrated that the response of wheat to nitrogen fertilization is heavily reliant on water condition. Previous studies suggest that water stress causes considerable senescence, and adverse effects on GY (Jamieson et al., 1995; Yang et al., 2000; Zhang and Yang, 2004), particularly when N supply exceeds the optimum value (Papastylianou, 1995; Karam et al., 2009). In our study, high rates of N application (N300 compared with N180) under rainfed conditions resulted in an average decrease in GY of 27.5 and 28.0% in 2012–2013 and 2013–2014, respectively. N fertilizer application should not be excessive in the drier year (2012–2013), causing a decrease in N accumulation in grains (GN) and resulting in a low grain yield and N harvest index (NHI; Table 4). Similarly, non-N application also had adverse effects on wheat production. Under non-N treatment, N mainly comes from atmospheric deposition and mineralization of soil organic matter (Dai et al., 2015). The minimum grain yield and AGN were also observed in the drier 2012–2013 growing season, mainly due to the decrease in soil N supply under dry conditions (only 59 kg ha^-1^ in our experimental site). Therefore, under dry conditions, non-N and high rates of N have a negative effect on wheat production, indicating the combined effect of water stress and deficient or excess N application.

Previous studies have also shown the effect of high fertilizer rates on reduced wheat root biomass and density in the subsoil (Hedegaard et al., 2006; Svoboda and Haberle, 2006; Rasmussen et al., 2015). In this study, compared with N180 treatment, higher N (N240 and N300) significantly decreased RWD in the 100 cm soil profile (Figure 2). Appropriate N application can improve the ability of wheat to absorb soil water and N in the deep soil layer during the growing season. N fertilizer application decreased the RWD maybe due to the effect on the R / S ratio, which decreased with increasing N application (Figure 4). The increase in R/S under water- and N-deficient conditions suggests that above–ground parts of wheat plants are more likely to be affected by water and N stress than the root system. The above–ground growth of wheat is often associated with root biomass accumulation (Anderson et al., 1998; Thorup-Kristensen et al., 2009; Barraclough et al., 2010). It has even been shown in the field studies that a high N application can reduce winter wheat root depth as well as density in the subsoil (Ruggiero and Angelino, 2011; Rasmussen et al., 2015).

Irrigation was also found to increase RWD compared to low- and non-irrigation treatments (Xue et al., 2003; Li et al., 2010). Under limited-irrigation treatment, RLD was higher than that under non-irrigation in the 0–80 cm soil layer at maturity (Barraclough and Leigh, 1984; Qin et al., 2004). According to Robertson et al. (1993), RLD increases with a decrease in available soil water from 100 to approximately 30%, followed by a significant decrease at less than 20%. In this study, irrigation caused an increase in RWD of 57.7 and 43.0% in the 100 cm soil profile in 2012–2013 and 2013–2014, respectively, and a corresponding increase of 48.7 and 42.5% in the 0–40 cm soil layer (Figure 3). Moreover, the combined effect of irrigation and N on root growth was also apparent, with irrigation (W2) plus N180 and N300 producing maximum and minimum RWD values in both growing seasons, respectively (Figure 2).

Root weight density was positively correlated with ET (Table 6). It has been suggested that optimal nutrient supply and mild soil water stress (65–70% of the field capacity) can cause an increase in WUE (Mandal et al., 2005; Zhang et al., 2006; Wang et al., 2012; Liu et al., 2016); however, in contrast, Karam et al. (2009) suggested that WUE is unaffected by both supplemental irrigation and N rate. In this study, ANOVA showed the effect of irrigation on WUE_DM_, but not WUE_Y_. Biomass WUE (WUE_F_, WUE_M_, and WUE_DM_) under irrigation conditions (W2) was lower than that under rainfed conditions (W0) in both growing seasons. N application increased WUE_F_ during vegetative growth stage, but higher N treatment (N240 and N300) had lower WUE_M_ for biomass during grain-filling (Table 3). Under field conditions, ET is divided into evaporation and transpiration (Youri et al., 2010), evaporation considered a non-productive component (Singh et al., 2011). Reduced evaporation could therefore play an important role in increasing the WUE of wheat (Liu et al., 2002). Increasing biomass before flowering stage reduced soil evaporation, thus offsetting increased loss through the plants (Wang et al., 2018), increasing biomass WUE through enhancing water use during vegetative growth under appropriate N rates.

Improved AGN synergistically increases grain yield and NUE in wheat (Foulkes et al., 2009; Sylvester–Bradley and Kindred, 2009). Optimizing the root system is therefore an important consideration in terms of improved NUE (Foulkes et al., 2009; Gaju et al., 2011). In this study, because available N is derived from both fertilizer and soil, increasing with increasing N application, the observed variation in UPE was thought to mainly be the result of fluctuations in AGN. N accumulation in leaves and grains was positively correlated with yield and WUE and NUE. Moreover, RWD was positively correlated with yield, NUE and ET at all sampling times (Table 6), suggesting that an increase in root biomass causes an increase in yield and NUE, but also resulting in rapid soil water consumption and lower WUE_DM_. The coefficients between RWD and yield, and RWD and NUE were higher at flowering than at jointing and maturity. This finding suggests that root growth and development at flowering is beneficial to yield and water and nitrogen use efficiencies in winter wheat.

## Conclusion

Irrigation caused an increase in wheat yield and RWD, but a decrease in WUE_DM_. Moreover, N application caused significant increases in grain yield and WUE, but decreases in NUE and RWD. Since RWD was positively correlated with GY, ET and NUE, it is also suggested that increases in root biomass cause an increase in NUE; however, a large root system also results in rapid soil water consumption. Overall, these findings suggest that increases in WUE and NUE are the result of optimization of the root system, which in turn contribute to the increase in grain yield. Therefore, N rate of 240 kg ha^-1^ with two irrigations can be recommend for winter wheat under the conditions in the North China Plain and those regions with similar conditions.

## Author Contributions

CW conceived of and designed the study. WL and JW analyzed the data and wrote the manuscript. WL, JW, and GM carried out the field measurements and root analysis. QW, HL, and YX critically reviewed the manuscript. DM and GK assisted with manuscript writing and editing. All authors approved the final version of the manuscript.

## Conflict of Interest Statement

The authors declare that the research was conducted in the absence of any commercial or financial relationships that could be construed as a potential conflict of interest.
